# Atropine in Heart Disease

**Published:** 1892-02

**Authors:** 


### ATROPINE IN HEART DISEASES.

DR. CARDARELLI (*Norsk Magazin for Loegevindenskaben*, No. 4, 1891) has found that atropine in doses of one-half to two milligrammes (1-128th to 1-32d of a grain) injected subcutaneously removes the inhibitory influence of the pneumogastric. The pulse increases in frequency and the blood pressure diminishes. Hence atropine is indicated in irritation of the pneumogastric. Whenever a slow pulse, with dizziness, convulsions and syncope, are present, atropine is indicated.—*The Medical and Surgical Reporter.*

